# Indirect Co-Culture of Testicular Cells with Bone Marrow Mesenchymal Stem Cells Leads to Male Germ Cell-Specific Gene Expressions

**DOI:** 10.22074/cellj.2019.5654

**Published:** 2018-08-07

**Authors:** Mehrdad Ghorbanlou, Alireza Abdanipour, Reza Shirazi, Nasim Malekmohammadi, Saeed Shokri, Reza Nejatbakhsh

**Affiliations:** 1 Department of Anatomical Sciences, School of Medicine, Zanjan University of Medical Sciences, Zanjan, Iran; 2 Department of Anatomical Sciences, School of Medicine, Iran University of Medical Sciences, Tehran, Iran; 3 Cellular and Molecular Research Center, Iran University of Medical Sciences, Tehran, Iran

**Keywords:** Co-Culture, Germ Cells, Mesenchymal Stem Cells, Retinoic Acid, Testis

## Abstract

**Objective:**

Non-obstructive azoospermia is mostly irreversible. Efforts to cure this type of infertility have led to the application of stem cells in the reproduction field. In the present study, testicular cell-mediated differentiation of male germ-like cells from bone marrow-derived mesenchymal stem cells (BM-MSCs) in an *in vitro* indirect co-culture system is investigated.

**Materials and Methods:**

In this experimental study, mouse BM-MSCs were isolated and cultured up to passage three. Identification of the cells was evaluated using specific surface markers by flow-cytometry technique. Four experimental groups were investigated: control, treatment with retinoic acid (RA), indirect co-culture with testicular cells, and combination of RA and indirect co-culture with testicular cells. Finally, following differentiation, the quantitative expression of germ cell-specific markers including *Dazl*, *Piwil2* and *Stra8* were evaluated by real-time polymerase chain reaction (PCR).

**Results:**

Molecular analysis revealed a significant increase in *Dazl* expression in the indirect co-culture with testicular cells group in comparison to the control group. Quantitative expression level of *Piwil2* was not significantly changed in comparison to the control group. *Stra8* expression was significantly higher in RA group in comparison to other groups.

**Conclusion:**

Indirect co-culture of BM-MSCs in the presence of testicular cells leads to expression of male germ cell-specific gene, *Dazl*, in the induced cells. Combination of co-culture with testicular cells and RA did not show any positive effect on the specific gene expressions.

## Introduction

Azoospermia has always been the most challenging issue associated with male infertility treatment (1). The common definition of azoospermia is the absence of sperm in the ejaculate (2). Causes of azoospermia can be classified in three categories: pre-testicular (related to endocrine diseases), testicular (internal diseases of the testis) and post-testicular (failure in ejaculation such as obstruction in reproductive ducts) (1). Non-obstructive azoospermia, which is caused by testis failure in producing sperm, involves 10% of infertile and 60% of azoospermic male (3). 

Efforts to treat the non-obstructive azoospermia have led to the application of stem cells in this field. Researchers have applied various sources of stem cells such as embryonic stem cells (4), induced pluripotent stem cells (iPSCs) (3), and mesenchymal stem cells derived from various sources such as bone marrow (5), umbilical cord (6), and adipose tissue (7). Different inducers such as bone morphogenetic proteins, especially BMP4 (8), retinoic acid (RA) (9), testosterone (10) and Sertoli-cell conditioned medium have also been used (11). 

Bone marrow stromal/mesenchymal stem/precursor cells (BM-MSCs) are considered as multipotent stem cells which have the potential of self-renewal and differentiation to different types of cell, such as osteocytes, chondrocytes and adipocytes (12). MSCs are easily accessible, expandable, immunosuppressive, and they do not elicit immediate immune responses; therefore they are a good choice for tissue engineering (13).

Primordial germ cells (PGCs) are the founder population of male germ cells originated from proximal epiblast and migrate through the dorsal mesentery to rich the developing gonads (5). These cells differentiate in a close relationship with the Sertoli cells and eventually generate spermatozoa. Differentiation of PGCs to spermatozoa occurs in different stages, while specific genes are expressed during this process (10). Deleted in azoospermia like (*Dazl*), Piwi like homolog 2 (*Piwil2*) and stimulated by RA gene 8 (*Stra8*) are three specific male germ cell genes expressed at different stages of male germ cells production (3, 5, 8, 10). *Dazl*, as a specific marker, is part of the Deleted in Azoospermia (DAZ) gene family which encodes RNA binding proteins necessary for germ cell development in different organisms (14). *Piwil2*, also known as *Mili*, is one of the three homologs of Piwi in mouse. *Piwil2* is present in adult germ cells, playing role in self-renewal of spermatogonial stem cells (15). *Stra8* is also a known molecular marker of spermatogonial stem cells inducing the beginning of meiosis (9). *Stra8* is expressed in adult seminiferous tubules at the time of mitosis-to-meiosis transitioning of male germ cells (16).

During spermatogenesis, different testicular cells -including germ, Sertoli, Leydig and peritubular myoid cells-interact with each other (17). Therefore, in the present investigation, testicular cells suspension is considered as an appropriate microenvironment and cocktail to induce derivation of germ cells from BM-MSCs. To enhance the induction, we also used RA, an active derivative of vitamin A.

In an indirect co-culture system, an insert filter with a biological microporous membrane is used which physically separates the upper compartment from the lower one, whereas it permits transfer of soluble factors through it (18). In this study, BM-MSCs were plated then the insert filter was applied and above the insert, the testicular cells –obtained from testis tissue digestion– were put. Finally, real-time PCR analysis was used for measuring quantitative abundance of *Dazl*, *Stra8* and *Piwil2* expressions in BM-MSCs. Our general purpose was preparing a condition in which male germ-cell specific genes can significantly be expressed in BM-MSCs.

## Materials and Methods

In this experimental study, Male Naval Medical Research Institute (NMRI) mice were housed under environmentally controlled conditions in 23-25°C and a 12/12 hours light/dark cycle. They were fed with a standard laboratory diet and accessed to drinking water ad libitum. Animals were treated in accordance with the Ethics Committee of Zanjan University of Medical Sciences (ZUMS.REC.1394.259, Zanjan, Iran). 

### Bone marrow mesenchymal stem cells isolation, culture and identification

Male NMRI mice of 4-6 weeks were sacrificed by cervical dislocation. Animals were soaked in povidone-iodine for 2-3 minutes, then two tiny incisions were made at the skin and superficial fascia of lower limbs. The lower limbs were removed with a pair of scissors separating it from the hip joint and put on a sterile gauze. The accompanied soft tissue (muscles, fasciae, and tendons) was removed, and femurs and tibiae were separated and put in a dish containing phosphate buffered saline (PBS, Gibco, Life Technologies, USA) and penicillin/streptomycin (Gibco, Life Technologies, USA). The dish was transferred under a laminar hood. The bones were subsequently washed again with PBS and put on a sterile gauze to dry. Both ends of the bones were cut, then with an insulin syringe containing high glucose Dulbecco’s Modified Eagle Medium (DMEM, Gibco, Life Technologies, USA) and 1% penicillin/streptomycin, all the contents of the bone’s lumen were flushed directly to 25 cm^2^ culture flask (SPL, life sciences, Korea) without any additional manipulation. The flushing was done several times, so that the lumen became pale. This method of collection of BM-MSCs is in accordance with Huang et al. (13). At first, BM-MSCs samples were cultured in DMEM supplemented with 10% fetal bovine serum (FBS, Gibco, life technologies, USA), 100 U/ml penicillin, and 100 mg/ ml streptomycin. The cells were then transferred to a 25 cm^2^ culture flask and incubated at 37°C and 5% CO_2_. After 48 hours non-adherent cells were removed by washing and replacement of the medium. The culture medium was changed every two days until the cells became 80% confluent. The cells were harvested with trypsin-EDTA 0.25% (Gibco, Life Technologies, USA) and passaged up to three times (P3). To identify BM-MSCs, surface antigens of the cells were evaluated by flow-cytometer. Concisely, cells at passage three were harvested and cell suspension was stained with fluorescence conjugated antibodies phycoerythrin-conjugated rat anti-mouse CD73, fluorescein isothiocyanate-conjugated rat anti- mouse CD44, phycoerythrin-conjugated rat anti-mouse CD90, fluorescein isothiocyanate-conjugated rat anti- mouse CD45 and phycoerythrin-conjugated rat anti- mouse CD34 (Abcam, USA) for 45 minutes at 4°C. Following the wash with PBS, staining buffer was used and cells were ready for flow-cytometry analysis. Cells were incubated by isotype control anti-bodies to measure nonspecific background signals. Flow-cytometry analysis was performed by BD FACsort device (BD Biosciences, USA).

### Testicular cells suspension preparation 

**Fig.1:**
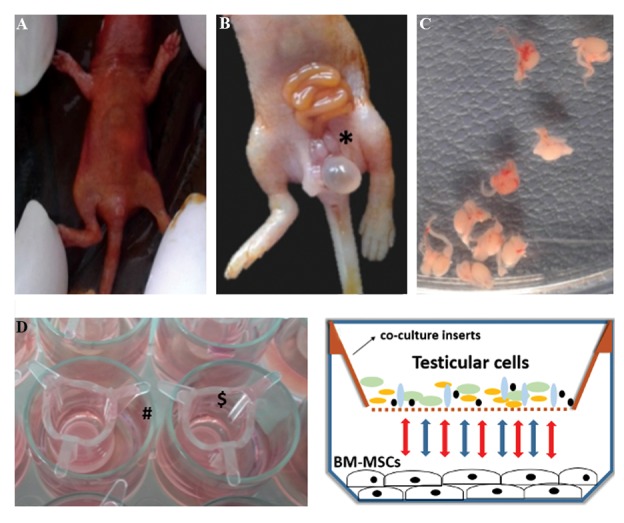
Testis isolation and indirect co-culture system. A. Sacrificed mouse (1-3 days old) was soaked in povidone-iodine, B. A tiny incision through skin, muscles and peritoneum is done in the lower abdomen region and by pushing the abdominal walls, intestinal loops, urinary bladder and testes (*) become visible, C. Isolated testes with accompanying tissues, D. Co-culture inserts ($) located on 24-well plate (#), and E. Schematic diagram showing interactions between the upper and lower compartments of indirect co-culture system.

Twelve male NMRI mice neonates (1-3 days old) were sacrificed. Mice were soaked in povidone-iodine for 2 minutes. Then a tiny cut through the skin, muscles and peritoneum were made at the lower part of the abdomen. By gently pressing the abdominal walls, intestinal loops, urinary bladder and accompanying testes were detectable. Intestinal loops and urinary bladder were set aside, so that we could see the tiny testes more clearly. By using a pair of tiny, sharp tip, sterile scissors testes were removed and put in a dish containing PBS and penicillin/streptomycin. The accompanying tissues of testis were removed and washed again with PBS. Testes were detunicated and smashed for enzymatic digestion in a 15 ml falcon (SPL, life sciences, Korea). Five milliliter of trypsin-EDTA was added to the falcon and vigorously shaken. It was subsequently left at room temperature for 3 minutes. After additional vigorous pipetting, the relatively homogenous solution was centrifuged at 300 g for 5 minutes. Then, the supernatant was removed and FBS was added to a total amount of 12 ml. After pipetting, testicular cell suspension was ready for co-culturing. This method of preparing testis tissue is a modified approach of Lacham-Kaplan et al. (19) study (Fig .1). 

### Indirect co-culture

BM-MSCs of passage three were plated (0.01×10^5^ cells/ well) in a 24-well plate (SPL, life sciences, Korea) before preparing the testicular cells suspension. When BM- MSCs were reached to 70% confluence (0.05×10^5^ cells/ well), the co-culture process was started. The 0.4 µm pore diameter insert filters (ThinCertTM cell culture insert for 24-well plates, Greiner Bio-One International, Australia) were located above the plates and then 0.5 ml of testicular cells suspension were applied over the filter (0.3×10^5^ cells/filter). Culture medium (DMEM plus 1% FBS) was added to a proper amount, so that both compartments could interact with each other. Every day for seven days, 200 µl of the medium was removed and fresh 200 µl medium was added. After 7 days of co-culture, BM- MSCs were removed from the plates by trypsin-EDTA for RNA extraction and real-time polymerase chain reaction (PCR) analysis.

### Experimental groups 

BM-MSCs at passage three were investigated in 4 groups: Control (3 separate 25 cm^2^ flasks with 80% confluence of BM-MSCs in passage 3 were cultured in DMEM plus 1% FBS); RA (3 separate 25 cm^2^ flasks with 80% confluence of BM-MSCs in passage 3 were cultured in DMEM containing 10 µmol/l RA plus 1% FBS for 7 days, RA was freshly added every other day); co-culture (BM-MSCs of passage 3 were plated in 12 wells of a 24well plate and 12 insert filters were located above them. Then 0.5 ml of testicular cells suspension was added above the filters and this indirect co-culture system was continued in DMEM plus 1% FBS for 7 days, every 4 wells were considered one separate sub-group, so we had 3 separate sub-groups in this group with the same condition); co-culture plus RA (the same condition as co- culture group, in addition to 10 µmol/l RA). 

### RNAisolation and real-time polymerase chain reaction analysis

Real-time PCR was carried out with cDNA from all experimental groups. Using Revert aid™ first strand cDNA synthesis kit (Fermentas, Germany), 1 µg purified RNA from cultured cells was used to synthesize 20 µl cDNA, according to the manufacturer’s instructions. cDNA was used to quantify *Dazl*, *Piwil2* and *Stra8* gene expression levels. As an internal control for normalization, ß-actin was used (19). The sequence of primers is presented in Table 1. The PCR reaction was performed in a 12.5 µl final volume (sense and anti-sense primers, cDNA and SYBR® Green I (Fermentas, Thermo Fisher Scientific, Inc.) and carried out for 40 cycles (StepOnePlus™ Real- Time PCR System, Thermo Fisher Scientific, Inc.). Delta Ct method was used for the analysis of relative changes in mRNA levels (20). 

### Statistical analysis

Data were analyzed by SPSS 16 software (SPSS, Inc., Chicago, IL, USA). All data are presented as means ± standard error of mean from 3 independent experiments. To compare differences of means in multiple tests, One- Way analysis of variance (ANOVA, post hoc Tukey) was used. Relative quantification method was applied for real- time PCR analysis. Values of P=0.05 were considered statistically significant. 

## Results

### Bone marrow-derived mesenchymal stem cells isolation and culture

**Fig.2:**
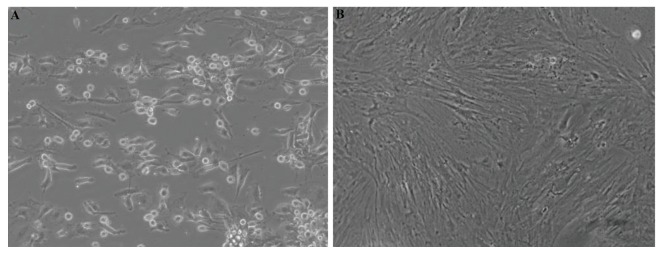
Representative photomicrographs of bone marrow-derived mesenchymal stem cells (BM-MSCs) in culture. A. Cell attachment of the freshly extracted BM-MSCs at 24 hours and B. BM-MSCs at passage 1 [scale bar 200 µm, ×400 magnification (inverted microscope, Nikon Eclipse Ti-S, USA)].

The results showed that BM-MSCs were attached to the dish surface, after 24 hours cultivation. After adhering, BM-MSCs were fibroblast-like shape (Fig .2A). By replacing the medium, majority of the non-adherent cells were eliminated and adherent cells gradually proliferated. After 8 days, the attached cells became confluent and could be sub-cultured (Fig .2B).

### Flow-cytometry analysis

**Fig.3:**
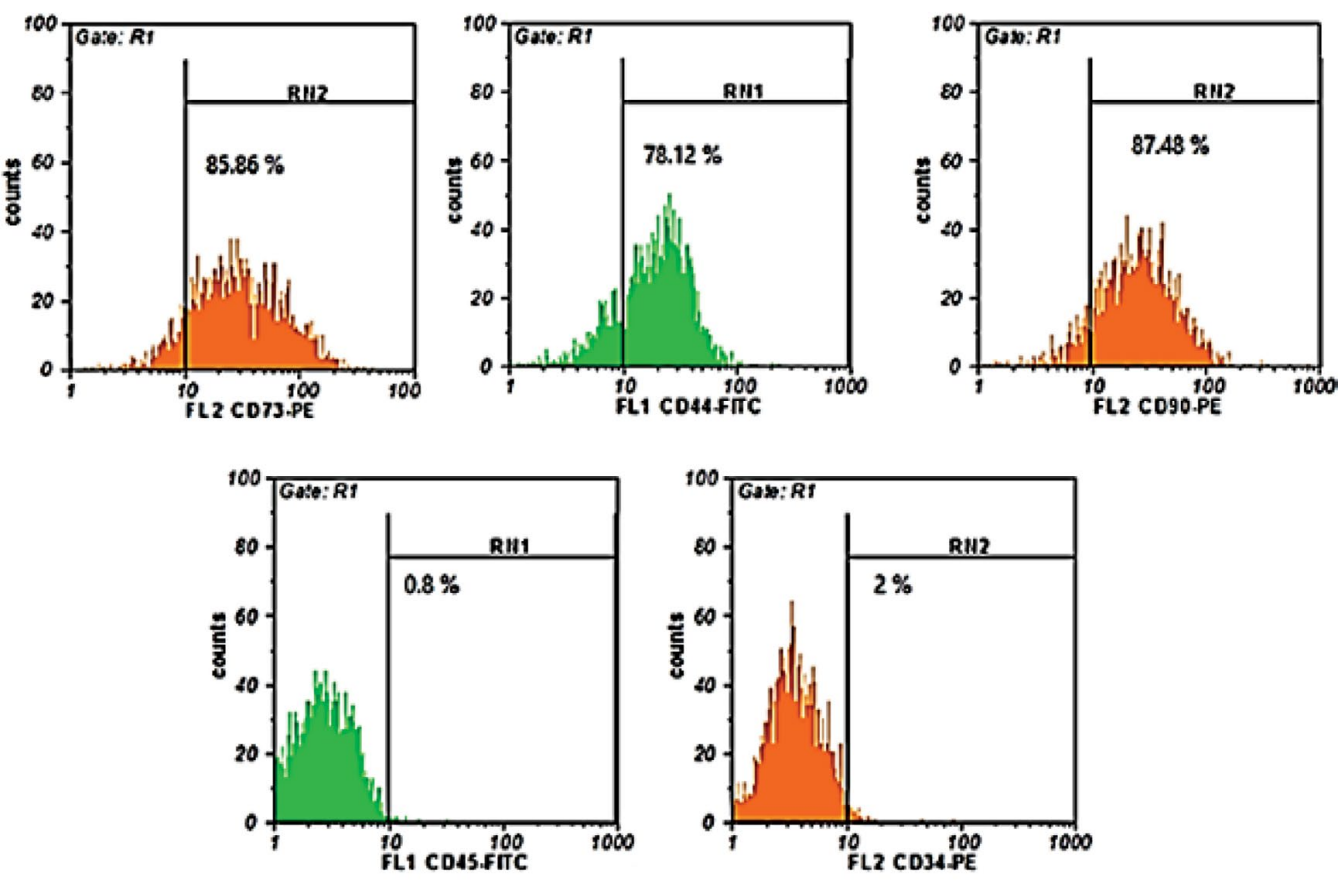
Detection of specific CD markers in BM-MSCs by flow cytometric analysis. Mouse mesenchymal stem cells were stained with fluorescence conjugated antibodies phycoerythrin-conjugated rat anti-mouse CD73 (85.86%), Fluorescein isothiocyanate-conjugated rat anti-mouse CD44 (78.12%), phycoerythrinconjugated rat anti-mouse CD90 (87.48%), Fluorescein isothiocyanate-conjugated rat anti-mouse CD45 (0.8%), and phycoerythrin-conjugated rat anti- mouse CD34 (2%).

BM-MSCs of passage three were analyzed for specific mesenchymal and hematopoietic markers using flow cytometry assay. Flow-cytometry results demonstrated that BM-MSCs of passage three are positive for CD73 (85.86%), CD90 (87.48%) and CD44 (78.12%), while it was negative for CD45 (0.8%) and CD34 (2%, Fig .3).

**Table 1:** Primer sequences and real-time polymerase chain reaction parameters. Primers for amplification of target sequences, and size of the fragment amplified are presented


Temperature (˚C)	Size	Primer sequence (5ˊ-3ˊ)	Primer

β-actin	F: GGTCATCACTATTGGCAACG	72	60
	R: ACGGATGTCAACGTCACACT		
Dazl	F: AAGGCAAAATCATGCCAAAC	133	60
	R: TCCTGATTTCGGTTTCATCC		
Stra8	F: CTCCTCCTCCACTCTGTTG	135	60
	R: GCGGCAGAGACAATAGGAAG		
Piwil2	F: CCTCCAGCTCTGTCTCCAAC	95	60
	R: CCTTGCTTGACCAAAAGCTC		


Primers were designed by Gene Runner software (Produced by: Pishgam Biotec. Co.).

### Gene expression

**Fig.4:**
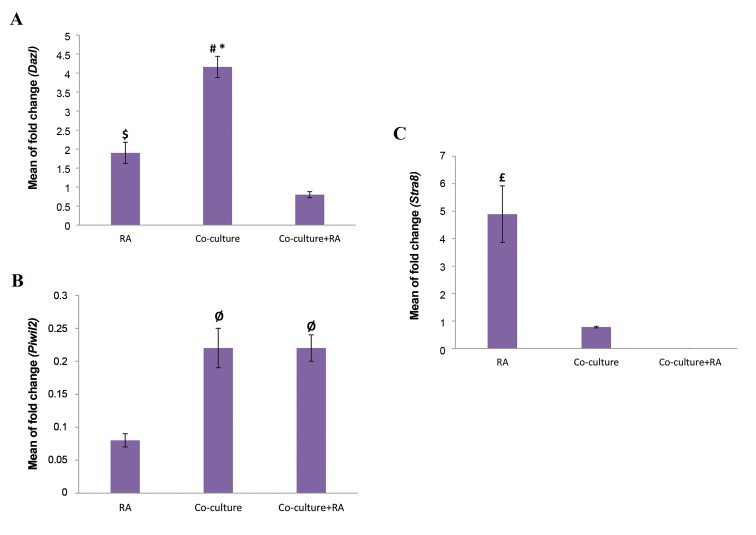
Relative experssion fold change of A. *Dazl*, B. *Piwil2*, and C. *Stra8*. Real-time polymerase chain reaction results are presented as relative gene expression normalized to ß-actin mRNA amplification. Significant increase of *Dazl* in co-culture group and *Stra8* in RA group are evident. The bars indicate the mean ± SEM. *; Co-culture vs. RA (P=0.01), #; Co- culture vs. co-culture+RA (P=0.001), $; RA vs. co-culture plus RA (P=0.01), Ø; Co-culture and co-culture+RA vs. RA (P=0.006), and £; RA vs. co-culture and co-culture plus RA (P=0.003).

The changes in expression of *Dazl*, *Piwil2* and *Stra8* in the different experimental groups were examined using quantitative reverse-transcription real-time PCR. The results are presented relative to control group (BM-MSCs cultured in DMEM plus 1% FBS) (Fig .4).

The mean value of fold-change for *Dazl* in the study groups was significantly increased in comparison to the control group. *Dazl* mRNA expressions in the co-culture group (4.16 ± 0.28) was significantly up-regulated when compared to RA (1.9 ± 0.28, P=0.01) and co-culture plus RA induction (0.8 ± 0.08, P=0.001). Furthermore, *Dazl* expression was significantly higher in RAgroup compared to co-culture plus RA group (P=0.01, Fig .4A).

*Piwil2* expression level in the study groups was not significantly increased in comparison with the control group (P>0.05), but it shows higher expression levels in co-culture (0.22 ± 0.03) and co-culture plus RA (0.22 ± 0.02) groups compared to RA group (0.08 ± 0.01, P=0.006, Fig .4B). 

The mean value of fold-change for *Stra8* in RA group was significantly increased in comparison with the co- culture and co-culture plus RA groups. *Stra8* mRNA expressions in the RAgroup (4.89 ± 1.03) was significantly up-regulated in comparison with co-culture (0.78 ± 0.03, P=0.003) and co-culture plus RA group (P=0.001). No expression of *Stra8* was detected in the co-culture plus RA group (Fig .4C). 

## Discussion

In order to differentiate BM-MSCs toward germ-like cells, in this study, testicular cells suspension was used as an effective microenvironment in an indirect co-culture system. For better induction of differentiation, synergic effect of RA and testicular cells was also investigated.

The results of this study indicated that indirect co- culture of BM-MSCs with testicular cells increased male germ cell-specific gene expression, *Dazl*. Utilizing RA could increase *Stra8* gene expression, considerably. Combination of RA and testicular cells did not show a positive effect on specific male germ cells genes expression.

BM-MSCs are commonly used in experimental studies, in terms of easy availability, isolation and culture. In addition, they are immunosuppressive and can be obtained from an adult person without ethical issues (12, 13). In this study the results of BM-MSCs culture and identification based on flow-cytometric assay were in accordance with the method of Huang et al. (13). In this particular method, BM-MSCs are isolated and cultured without additional manipulation. Therefore BM-MSCs are cultured in their primary niche to permit better survival and growth rate.

The relation of cell culture environments to what really happens in an organism can be enhanced by creating microenvironments behaving similar to *in vivo* condition (21). Therefore, testicular cells suspension was used to improve microenvironment and generate inductive factors, such as bone morphogenetic protein 4 (BMP4) (22), stem cell factor (SCF) (23), leukemia inhibitory factor (LIF) (24) and insulin-like growth factor I (IGF-I) (25), for BM-MSCs differentiation toward male germ-like cells. Somatic cells of testis affect the spermatogenesis and the differentiation process of germ cells through interactions with each other. In the testis tissue, Sertoli cells are in direct contact with germ cells, but interstitial Leydig cells, macrophages and peritubular myoid cells affect Sertoli cells, so they indirectly influence the germ cells (26). Sertoli cells are in charge of producing factors for metabolism of germ cells, such as lactate, transferrin and androgen binding proteins and they are also responsible for producing regulatory factors, such as stem cell factor, transforming growth factors of α and ß, insulin like growth factor-I (IGF-1) and some others. IGF-1 has a receptor on germ cells and it functions in maintaining and regulation of DNA synthesis. Sertoli cells also possess the follicle stimulating hormone (FSH) receptor, after binding of FSH hormone on its receptor, cAMP levels in sertoli cells will be increased leading to the activation of phosphoinositide 3-kinase pathway, as a supporter of germ cells differentiation (27). Sertoli cells possess androgen receptors as well, therefore androgen influences their function and eventually the spermatogenesis. Interstitial Leydig cells also possess androgen receptors and steroidogenesis occur when androgen binds to its receptors on the Leydig cells. The peritubular myoid cells produce a paracrine factor which modulates the function of Sertoli cells (PMODS), so it affect spermatogenesis indirectly (28). Since all of the testicular cells interact with each other and they are necessary for spermatogenesis, we chose a method of preparing testis tissue which keeps all of the testicular cells in the co-culture system. Our method was in accordance with the protocol of Lacham- Kaplan et al. (19). 

RA is small, polar molecule which easily passes the tissues and induces its action by binding to retinoid receptors on nuclei (29). Binding of RA to nuclear retinoid and rexinoid receptors on nuclei of spermatogonia increases the expression of transcription factor *SALL4A*. This leads to higher expression of receptor tyrosine kinase (4) which is essential for spermatogonia differentiation (30). Studies on differentiation of stem cells to male germ cells have used RA as an inducer (5, 9, 10, 30). In this study, we have applied the 10 µmol/l concentration of RA which is in accordance with the other studies (5, 31). 

*Dazl* is an important gene in differentiation and development and it may function as a master gene in germ cell differentiation (32). In a recent study (33), differentiation of BM-MSCs of goat to germ cell-like cells has been done by overexpression of *Dazl*, Boule and *Stra8*. In the present study, *Dazl* expression is significantly increased by RA induction and indirect co- culture with testicular cells in comparison to the control group. Combination of RA and indirect co-culture with testicular cells did not lead to significant results in *Dazl* expression. Geens et al. (34) also could not observe better specific gene expression in human embryonic stem cells when they combined Sertoli cell conditioned medium with BMP4 for better induction. Increased expression of *Dazl* by the RA induction has been reported in other studies (5, 6, 8-10, 31). Silva et al (10) has reported a temporal expression of *Dazl* in embryonic stem cells treated with RA, its expression is initially low and by time it is increased. We might have observed a higher quantitative gene expression if we continued the process of treatment and indirect co-culture. *Piwil2* expression level did not show any significant change after 7 days of RA induction and indirect co-culture. It seems that temporal expression of *Piwil2* is in a way which is decreased after 7 days of RA induction, it is evident in the study of Silva et al. (10). In their study embryonic stem cells at 7 days post-treatment by RA, showed the least expression rate of *Piwil2*, however higher expression rates were evident when they continued the treatment. Higher expression levels of *Piwil2* are evident in days of 2 and 4 of RA induction, as well. Probable higher *Piwil2* expression can be expected if we continue RA treatment for more than 7 days (5, 10). Although several studies have reported *Piwil2* as a mitotic gene, there are other studies reporting higher expression of this gene in spermatids and spermiogenesis (35) and indicating the impaired spermiogenesis in mice lacking *Piwil2* gene (36). Therefore, incomplete differentiation of BM-MSCs toward germ cells, can be a probable reason why *Piwil2* expression is low in this study. Since *Stra8* is the target gene of RA (3), as expected, significant expression rate of this gene is observed after 7 days of RA treatment. Other studies have reported *Stra8* expression when stem cells are treated with RA (5, 9). Simultaneous application of RA and co-culture with testicular cells could not lead to significant *Stra8* expression.

The combination of RA and testicular cells could not lead to better differentiation of BM-MSCs. It is hypothesized that RA is mostly absorbed by the spermatogonial cells of the testis, bearing RA receptors (37). Cytochrome P450, family 26, subfamily b, polypeptide 1 (CYP26B1) enzyme is present in spermatogonia cytoplasm, degrading RA into metabolites, some of which are inactive (38). This enzyme is present in testes of newborn mice which inserts an inhibitory effect on spermatogonial differentiation by inhibiting the expression of *Stra8* (39). So, the presence of newborn testicular cells in the co-culture system can probably inhibit *Stra8* expression, as we can see in our study. The absorption of RA by testicular cells and the inhibitory effect of CYP26B1 are probable reasons why the combination of RA and testicular cells could not lead to better differentiation of BM-MSCs. 

Although differentiation of male germ cells occurs in direct contact with Sertoli cells (37), endocrine and auto/ paracrine factors affect the process of differentiation (11). In the present study, the endocrine and paracrine signaling, provided by testicular cells, was the source of BM-MSCs differentiation. The microporous barrier of insert filters do not allow the cells to pass, but the factors secreted by testicular cells can pass through the pores and induce differentiation of BM-MSCs, since both compartments share a common culture medium. Although some studies consider the direct contact as an essential element for differentiation, other studies have reported the expression of specific genes is sufficient without direct contact (40).

The novelty of this study is related to indirect co-culture of testicular cells with BM-MSCs in the presence of RA. Our hypothesis was that different testicular cells and their interactions promote the Sertoli cells function and they may serve as a proper microenvironment for induction of differentiation in BM-MSCs toward male germ like cells. 

## Conclusion

Indirect co-culture of testicular cells with BM-MSCs leads to significant increase in male germ cell specific gene *Dazl*. RA is effective to increase the gene expression, but combined effect of co-culture and RA does not increase the specific male germ cells genes expression.
